# A Mechanical Analysis of Chemically Stimulated Linear Shape Memory Polymer Actuation

**DOI:** 10.3390/ma14030481

**Published:** 2021-01-20

**Authors:** Hakan Dumlu, Axel Marquardt, Elias M. Zirdehi, Fathollah Varnik, Yucen Shen, Klaus Neuking, Gunther Eggeler

**Affiliations:** 1 Institute for Materials (IFM), Ruhr University Bochum, Universitätsstr. 150, 44801 Bochum, Germany; axel.marquardt@rub.de (A.M.); yucen.shen@rub.de (Y.S.); klaus.neuking@rub.de (K.N.); gunther.eggeler@rub.de (G.E.); 2 Interdisciplinary Centre for Advanced Materials Simulation (ICAMS), Ruhr University Bochum, Universitätsstr. 150, 44801 Bochum, Germany; elias.mahmoudinezhadzirdehi@rub.de (E.M.Z.); fathollah.varnik@rub.de (F.V.)

**Keywords:** shape memory polymers, ESTANE ETE 75DT3, influence of solvents on glass temperature, chemically triggered actuation, actuator performance

## Abstract

In the present work, we study the role of programming strain (50% and 100%), end loads (0, 0.5, 1.0, and 1.5 MPa), and chemical environments (acetone, ethanol, and water) on the exploitable stroke of linear shape memory polymer (SMP) actuators made from ESTANE ETE 75DT3 (SMP-E). Dynamic mechanical thermal analysis (DMTA) shows how the uptake of solvents results in a decrease in the glass temperature of the molecular switch component of SMP-E. A novel in situ technique allows studying chemically triggered shape recovery as a function of time. It is found that the velocity of actuation decreases in the order acetone > ethanol > water, while the exploitable strokes show the inverse tendency and increases in the order water > ethanol > acetone. The results are interpreted on the basis of the underlying chemical (how solvents affect thermophysical properties) and micromechanical processes (the phenomenological spring dashpot model of Lethersich type rationalizes the behavior). The study provides initial data which can be used for micromechanical modeling of chemically triggered actuation of SMPs. The results are discussed in the light of underlying chemical and mechanical elementary processes, and areas in need of further work are highlighted.

## 1. Introduction

Shape memory polymers (SMPs) are stimulus-responsive materials which show shape recovery. There are excellent reviews which cover this fascinating class of materials (e.g., [1,2,3,4,5,6,7,8,9]). SMPs receive special attention in the biomedical field [1,2,8,9,10,11,12,13,14]. A few articles discussed potential applications in aerospace technology [15,16]. Sensor applications have also been considered [17]. Fundamental studies have focused on the interaction of shape recovery and swelling [18] and on effects associated with thermal cycling [19]. The activation of the one-way effect in SMPs by chemical stimuli has received increasing attention in the last decade [20,21,22,23,24,25].

**Figure 1:**
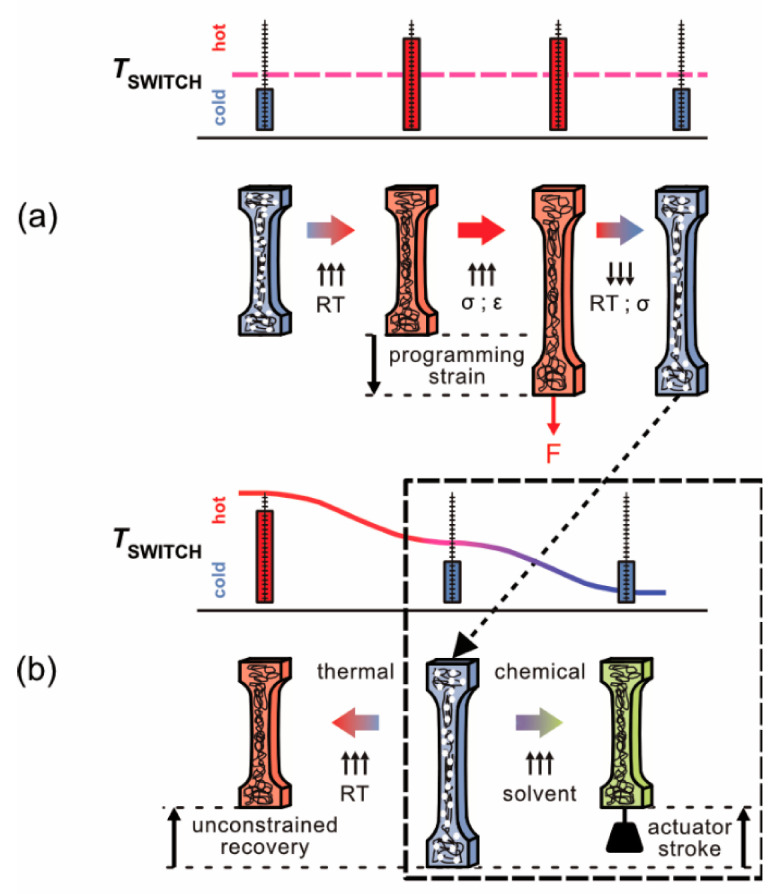
Shape memory polymer (SMP) programming and one-way effect. (**a**) Storage of entropic energy in a sequence consisting of heating to a temperature above the glass temperature of the molecular switch (*T*_SWITCH_), imposing the programming strain at this temperature, and constrained cooling of the material below *T*_SWITCH_. (**b**) Thermal (left) and chemical (right) triggering of the one-way effect. Thermal recovery is unconstrained (no end load). The chemically triggered one-way effect is exploited to lift an end load (actuation).

They consist of macromolecular networks with a suitable switching structure, which allows storing and recovering large strains. They are processed into a permanent original shape by extrusion or injection molding. SMPs can consist of block copolymers, one elastomer and another, with an appropriate glass temperature which acts as a molecular switch. During programming, the material is heated above the glass temperature of the molecular switch (*T*_SWITCH_). The programming strain is then imposed, and the molecular chains of the elastomer are stretched, thus increasing the entropic energy of the system. The constrained material is then cooled to room temperature and entropic/mechanical energy is stored (Figure 1a). It is today well established that programmed SMPs can recover their original shapes in several ways. Upon heating above *T*_SWITCH_, the elongated molecular chains can contract into their stable high-entropy configuration (left side of Figure 1b). Alternatively, the recovery process can be triggered by solvents which diffuse into the material and affect its morphology such that *T*_SWITCH_ decreases, which eventually allows the elongated macromolecular chains to contract (right side of Figure 1b).

The present work focuses on chemical activation (dashed rectangle in Figure 1b) and has two objectives. First, as part of a collaborative research program, it explores thermal–mechanical–chemical interactions in structural and functional engineering materials [26]. In this research activity, quantitative experimental results are required which can be used as input and/or as benchmark data for modelers from the fields of molecular dynamics [27,28,29] and continuum mechanics [30]. It turned out that, while there is a good understanding of the elementary molecular processes which govern the fascinating behavior of SMPs, no precise data exist which can be directly used for modeling of scale bridging materials. A model system was chosen consisting of a polyether-based thermoplastic polyurethane, abbreviated as SMP-E throughout this work, and three solvents: acetone (C_3_H_6_O), ethanol (C_2_H_6_O), and water (H_2_O). As a prerequisite for the present study, molecular mobilities of these solvents in SMP-E were measured and documented [31]. The second objective of the present work is to study the performance of SMP-E as a chemically triggered linear actuator. Linear actuators convert energy into straight-line motions, for positioning applications, typically with push-and-pull functionality [32]. A typical use is the control of various valves. In the case of SMP actuators, which operate in harsh chemical environments, it is important to understand how they are affected by the uptake of solvents from the environment.

The present study considers the one-way effect in chemically triggered linear SMP-E actuators. We use dynamic mechanical thermal analysis (DMTA) to investigate how *T*_SWITCH_ evolves when SMP-E takes up one of the solvents (acetone, ethanol, and water). Two programming states (imposed strains: 50% and 100%) are considered, and shape recovery triggered by the uptake of solvents (acetone, ethanol, and water) is investigated, studying both unconstrained and constrained shape recovery. Constrained recovery is investigated for increasing end loads, corresponding to stresses of 0.5, 1.0, and 1.5 MPa. In the light of previous results reported in the literature, the results are rationalized in terms of chemical changes associated with solvent uptake. Implications of these findings for chemically triggered SMP actuator operation are worked out, and evidence for their validity is demonstrated.

## 2. Materials and Methods

**Figure 2:**
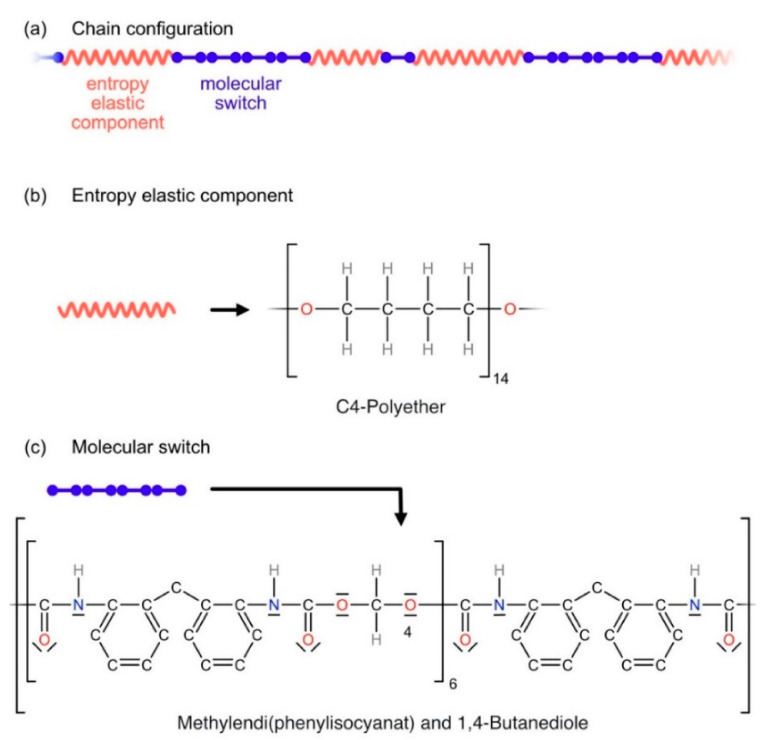
Schematic illustration of macromolecular structure of SMP actuators made from ESTANE ETE 75DT3 (SMP-E). (**a**) Macromolecular chain with two components: an entropy elastic component (red) and a molecular switch (blue). (**b**) Structural formula of elastic component. (**c**) Structural formula of molecular switch.

*SMP-E, injection molding and specimen geometries:* In the present work, we investigate ESTANE ETE 75DT3, which was purchased as granulate from Lubrizol Corp., Wickliffe, OH, USA [33]. Throughout the present work, we refer to this material as SMP-E. For analysis, the granulate was sent to a certified test lab for chemical analysis [34] and for measuring the distribution of molecular weights [35]. Gel permeation chromatography was used to determine the distribution of the molecular weights of the macromolecular chains of the polymer. The average molecular weight of the macromolecules was obtained as 132,237 atomic mass units, corresponding to a molar weight of 132 kg/mol. The chemical composition in weight percentage was analyzed as 52.6 wt.% methylendiphenylisocyanate (MDI), 29.6 wt.% C_4_-polyether, 15.1 wt.% 1,4-butandiole, and traces of dipropylenglycol and 1,3-butandiol (similar molecular structures as 1,4-butandiole). SMP-E is a polyether-based thermoplastic polyurethane [33]. Knowing how polyurethanes are synthesized [36], it is clear that the compounds of SMP-E form only one type of chain; therefore, it consists of macromolecular chains made up of two components. Figure 2 schematically illustrates the macromolecular structure of SMP-E.

**Figure 3:**
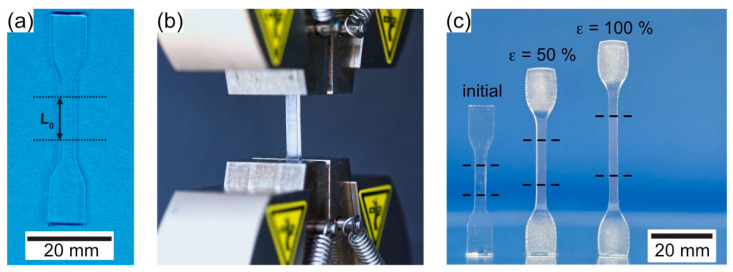
Specimen geometry and programming. (**a**) Dog-bone-type flat tensile specimen used in the present work. (**b**) Specimen mounted into test rig with heating chamber prior to processing. (**c**) As-processed specimen together with two specimens which were programmed imposing 50% and 100% programming strain.

Specimens were produced using injection molding, the processing details of which were described previously [37,38,39]. The granulate was dried in an Arburg Thermolift system from Lossbach, Germany. Plates of dimensions 57.5 × 66.5 × 2.1 mm^3^ were injection-molded using an Arburg Allrounder 270 M from Lossbach, Germany. From the extruded plates, dog-bone-type specimens, as recommended by the German standard for testing of polymers DIN 53504 [40], were cut out. The specimens had a width of 4 mm and a thickness of 2.1 mm. Their weight was close to 0.8 g. The specimen had a gauge length *L*_0_ of 10 mm (Figure 3a). Figure 3b shows the specimen mounted into the test rig of type Zwick/Roell Z2.5 equipped with a heating chamber (Mytron—Bio- und Solartechnik GmbH, Heilbad Heiligenstadt, Germany).

Details of programming were published previously [37,38,39], and it was also shown that the SMP-E specimens exhibit the one-way effect, as demonstrated in the left part of Figure 1b. It was also shown that programming can be repeated up to 50 times without losing actuator efficiency [37]. In the present work, programming was performed at 353.15 K (i.e., 80 °C), and two programming strains, 50% and 100% (corresponding to displacements Δ_PROG_ of 5 and 10 mm, respectively), were imposed, prior to constrained cooling. An as-processed and two programmed specimens (imposed programming strains 50% and 100%) are shown in Figure 3c.

**Figure 4:**
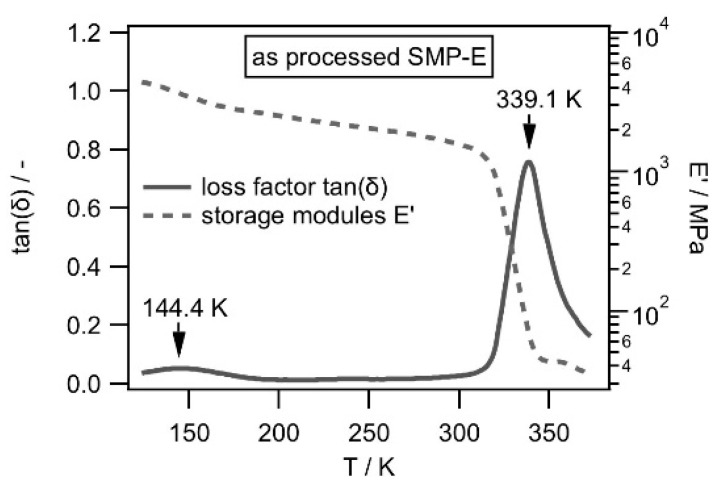
Dynamic mechanical thermal analysis (DMTA) results obtained for the as processed SMP-E.

*Dynamic mechanical thermal analysis:* In the present work, we performed dynamic mechanical thermal analysis [41,42,43,44] to determine the glass transition temperature of the molecular switch component of SMP-E. During a DMTA experiment, a set of parameters are continuously measured. One is the complex elastic modulus |E*| which has two components, the storage (*E*’) and the loss modulus (*E*″) [41,42,43,44], which stand for the elastic (*E*’) and the viscoplastic (*E*″) polymer properties. The other parameter, which is monitored during the experiment, is the loss factor tan(*δ*), which is given by tan(*δ*) = *E*″*/E*’. It is well-known textbook knowledge that, in the case of polymers, tan(*δ*) has a maximum at the glass transition temperature, where the material loses its mechanical integrity. It is also well known that *δ* represents the phase shift between the imposed strain and the resulting stress. In the present work, DMTA was performed and displacement/strain was controlled using a Gabo Eplexor 500 N test system from Netzsch Gabo Instruments GmbH, Ahlden, Germany. Small sinusoidal cyclic displacements corresponding to 0.1% strain were imposed at a constant frequency of 10 Hz while the temperature continuously increased from 123.15 K (i.e., −150 °C, rate of temperature ramp: 2 K/min). To keep the system aligned, a small end load of 1 N was applied. We described all details of our DMTA procedure in a previous publication [45]. The DMTA results in Figure 4 show how the storage modulus *E*’ and the loss factor tan(*δ*) of SMP-E depend on temperature. During the experiment, subsequent mechanical cycles were imposed in small 2 K intervals.

Figure 4 shows that there were two tan(*δ*) peaks, one at 144.40 K (i.e., −128.7 °C) and another at 339.10 K (i.e., 65.9 °C). Two loss peaks are typical for polymers with two components [43,44]. The much smaller (logarithmic scale) loss peak at 144.40 K can also correspond to a solid-state transition such as the well-known β and γ transitions [43]. As the material warms up, its free volume increases, and this promotes localized bond movements (bending and stretching). As can be seen, the effect of this transition on the storage modulus was very small. Important for the objectives of the present work is the peak at 339.10 K (i.e., 65.9 °C), which represents the temperature *T*_SWITCH_, where the second component of the macromolecular SMP-E chain loses its mechanical integrity.

The present work focuses on chemically triggered unconstrained and constrained recovery. After cutting and prior to chemical exposure, the tensile specimens were kept dry in a vacuum desiccator at 294.15 K (i.e., 21 °C, no light exposure) in order to avoid uncontrolled uptake of solvents. Chemical exposure was performed in a reaction vessel filled with acetone, ethanol, or water at 294.15 K, following the procedure outlined in [31,39]. After chemical exposure, the specimens were encapsulated into a glass tube and kept at 294.15 K for 2 weeks in order to establish chemical homogeneity. Our previously published diffusion data [31,39] allowed selecting appropriate exposure times (acetone: 1080 s to 90,000 s, ethanol: 86,400 s to 518,400 s, and water 1,368,000 s). In an attempt to establish comparable material states, target solvent concentrations between 1.3 × 10^−3^ and 6.5 × 10^−3^ mol/g were preliminary envisaged. This resulted in the exposure times listed in Table 1.

During these exposure times, SMP-E took up solvents. Their concentrations were measured by evaluating weight gains, two times per material state. The initial mass of the specimen $m_{0}$ and the mass after chemical exposure $m_{\mathrm{CH}}$ were measured. In order to check whether solvent evaporation occurred during DMTA experiments, masses $m_{\mathrm{DMTA}}$ were also determined after thermomechanical experiments. With the molecular weight $M_{S}$, the solvent concentrations $c_{s}$ which characterize the material states were obtained as

(1) $$c_{s}=\;\left(\left(m_{\mathrm{DMTA}}-m_{0}\right)/M_{S}\right)/m_{0}.$$

As can be seen from the data listed in Table 2, the maximum scatter when an experiment was repeated twice was of the order of 5% (observed for acetone; negligible for ethanol and water).

**Figure 5:**
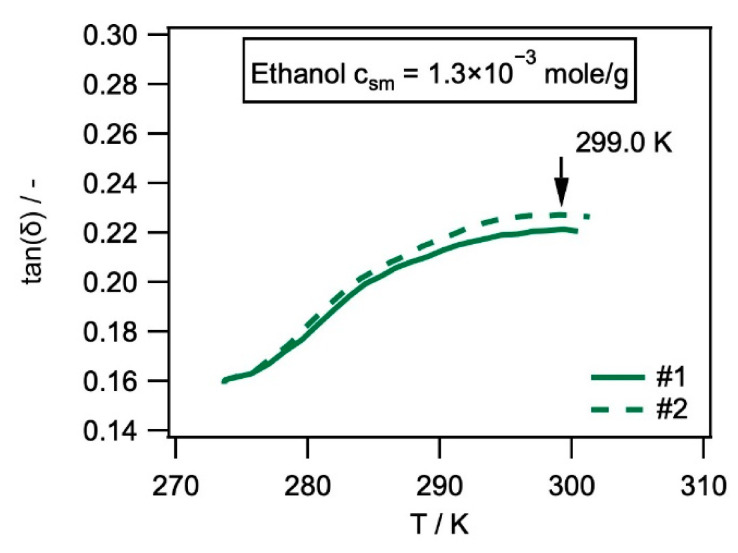
tan(*δ*) as a function of temperature (rate of temperature ramp: 2 K/min, frequency: 10 Hz, imposed strain amplitude: 0.1%, end load: 1 N) from two experiments performed for SMP-E with an ethanol concentration of 1.3 × 10^−3^ mol/g. The arrow indicates the position of *T*_SWITCH_ (identical in the two experiments shown).

SMP-E specimens with different solvent concentrations *c*_s_ were thermomechanically analyzed in order to determine *T*_SWITCH_. First, one screening experiment was performed for each material state over the whole temperature range, which provided a first indication of the position of *T*_SWITCH_ (data used for experimental guidance). This information was then used to perform two experiments per material state, in order to measure the exact position of *T*_SWITCH_. These experiments started 30 K below the loss peak observed in the first screening experiment, thus avoiding eventual evaporation losses during dynamical testing at lower temperatures. The two weight results for *m*_DMTA_ listed in Table 2 were obtained from these two experiments. It was found that, in all cases, these two experiments per material state yielded reproducible tan(*δ*)(*T*) curves, as shown for SMP-E with an ethanol concentration of 1.3 × 10^−3^ mol/g in Figure 5.

**Figure 6:**
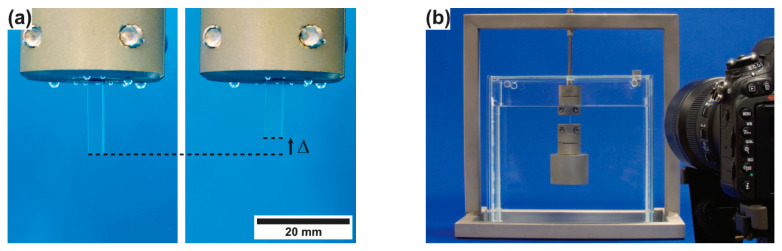
Experimental procedure to measure chemically triggered recovery. (**a**) Measurement of unconstrained recovery using rectangular specimens. Bubbles below grip indicate that specimen was induced in water. Left image: SMP-E specimen programmed to 50% exposed to water. Right image: Same specimen after 345,600 s (i.e., 96 h) of exposure. Black arrow indicates direction of positive deformation. (**b**) Documenting constrained recovery. SMP-E specimen programmed to 50% with end load (corresponding to 1.0 MPa) exposed to water at the beginning of the experiment. For quantitative evaluation, several hundred images were taken for each experiment in appropriate time intervals.

*Analysis of constrained and unconstrained recovery:* The key experiments performed in the present work consist of measuring the chemically triggered back-deformation at a constant temperature of 294.2 K (i.e., 21 °C). Figure 6a shows the unconstrained shape recovery of SMP-E programmed to 50% in water (the presence of air bubbles below the grip proves that the photograph was taken from an immersed specimen). The image on the left shows the specimen right after start of exposure to water. The image on the right was taken after 345,600 s (i.e., 96 h) exposure time, where unconstrained recovery resulted in a back-deformation toward the geometry prior to programming of Δ. We count this deformation as positive, when the lower part of the specimen moves up (shortening of the specimen) as shown in Figure 6a, and we refer to the time dependent displacement which accumulated during thermal exposure as Δ(*t*). Figure 6b documents how constrained recovery was measured in the presence of an end load. Experiments with three end loads were performed corresponding to uniaxial stresses of 0.5, 1.0, and 1.5 MPa. Figure 6b shows an SMP-E specimen programmed to 50% in water under a stress of 1.0 MPa at the beginning of a test. In order to monitor the evolution of Δ, several hundred photographs were taken in each experiment in time intervals of 300 s (i.e., 5 min, acetone), 1800 s (i.e., 30 min, ethanol), and 3600 s (i.e., 1 h, water). The duration of each experiment was between 86,400 s (i.e., 24 h, exposure to acetone) and 1,036,800 s (i.e., 288 h, exposure to water). 

**Figure 7:**
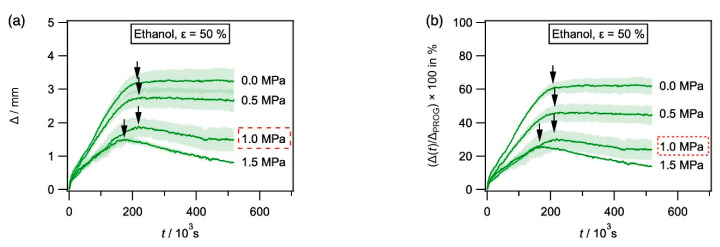
Evolution of Δ with time for SMP-E specimens programmed to 50% and exposed to ethanol (unconstrained and with varying end loads). (**a**) Δ in mm plotted as a function of time in s. (**b**) (Δ(*t*)/Δ_PROG_) × 100 in % vs. time in s.

Figure 7 shows how unconstrained and constrained recovery experiments were evaluated, using SMP-E programmed to 50% exposed to ethanol as an example. In Figure 7a, the displacement Δ(*t*) in mm is plotted as a function of time in s. This plot provides information on the actual displacements associated with chemical exposure. Figure 7b shows the same data plotted as (Δ(*t*)/Δ_PROG_) ×100 in % vs. time in s. This plot indicates the percentage of programming strain that can be recovered during chemical exposure. Each experiment was performed three times, and the results are presented as an average curve together with the observed scatter band (± absolute mean deviation from mean value). In Figure 7a, arrows indicate where and when the maximum recovery was reached. 

**Figure 8:**
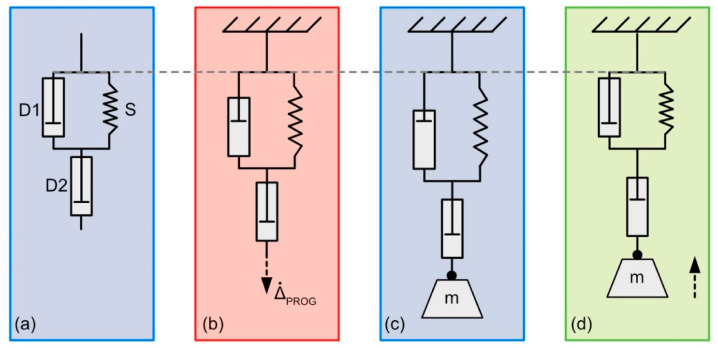
Schematic illustration of Lethersich [48] model. Temperature and chemistry color coding as introduced in Figure 1. (**a**) Model elements S, D1, and D2. (**b**) Programming. Application of a constant strain rate ${\dot{\Delta }}_{\mathrm{PROG}}$ in mechanical test rig (experiment: Figure 3b). (**c**) State after programming and prior to chemical exposure (experiment: Figure 3c). (**d**) Chemically triggered actuation (experiment: Figure 6b).

*Modeling the strain evolution in programmed SMP-E exposed to ethanol:* The time-dependent mechanical behavior of polymers is often described by rheological models [46,47]. In order to interpret the mechanical response of our SMP-E to chemical exposure, we used a model which was originally considered by Lethersich to discuss the deformation of bitumen [48]. The model without end load is shown in Figure 8a. It consists of a Voigt model (a Hookean spring S parallel to a Newtonian dashpot D1), which is in series with a second Newtonian dashpot D2. Hooke’s law describes the mechanical behavior of the spring element as

(2) $$\sigma_{S}=E_{S}\;\times \;\varepsilon_{S},$$

where $\sigma_{S}$ is the stress acting on the spring, $E_{S}$ is its spring constant, and $\varepsilon_{S}$ is the strain acting in the spring. We assume that the two dampers show Newtonian flow,

(3) $${\dot{\varepsilon }}_{D1}=\frac{1}{\eta_{D1}}\;\times \;\sigma_{D1}\;\mathrm{and}\;{\dot{\varepsilon }}_{D2}=\frac{1}{\eta_{D2}}\;\times \;\sigma_{D2},$$

where ${\dot{\varepsilon }}_{D1}$ and ${\dot{\varepsilon }}_{D2}$ are deformation rates, and $\eta_{D1}$ and $\eta_{D2}$ represent the viscosities of the two dashpot elements. $\sigma_{D1}$ and $\sigma_{D2}$ are the stresses which govern viscous flow in the two dashpots, $\sigma_{D1}$ results from the elongation of the spring, and $\sigma_{D2}$ represent the stress $\sigma_{m}$ associated with the end load.

In Figure 8a, at ambient temperature, the SMP-E component which represents the molecular switch, D1 in our model, is in a glassy state, which can resist mechanical loading. During programming at higher temperatures, above *T*_SWITCH_, the molecular switch adopts a liquid-like configuration and loses mechanical integrity. This allows imposing the programming strain at a constant displacement rate ${\dot{\Delta }}_{\mathrm{PROG}}$ of 30 mm/s (Figure 8b). The macromolecular chains of the entropy elastic component are stretched. The state after programming and cooling back down to ambient temperature, prior to chemical exposure at room temperature with an end load of a mass m, is shown in Figure 8c. Note that the spring *S* and the element D1 are elongated (equal displacements in the Voigt model), and programming has also caused some deformation of element D2. Figure 8d schematically illustrates chemical actuation, where the end load is lifted upward (dashed arrow). Only a part of the imposed programming strain is recovered, while another part represents Newtonian flow of element D2. Knowing that $\varepsilon_{S}=\;\varepsilon_{D1}$ and that the total strain is given by $\varepsilon_{\mathrm{tot}}=\;\varepsilon_{D1}+\varepsilon_{D2}$, it is easy to numerically calculate the evolution of the total strain of our system under an end load $\sigma_{m}$ with time (knowing the internal stress $\sigma_{S}$, which was introduced by the part of the programming strain $\Delta_{\mathrm{PROG}}^{*}$ which elongates the spring). In one time step Δ*t*, the dashpot D1 contracts by

(4) $$\Delta \varepsilon_{D1}=\;c_{D1}\;\times \;(\sigma_{s}+\;\sigma_{m})\;\times \;\Delta t,$$

and the stress acting on the spring decreases. At the same time, the dashpot D2 elongates driven by the end load.

(5) $$\Delta \varepsilon_{D2}=\;c_{D2}\;\times \;\sigma_{m}\;\times \;\Delta t.$$

When considering the programmed state as the initial condition $\varepsilon_{tot}\left(t\right)=0$, one can obtain the evolution of strain with time as

(6) $$\varepsilon_{\mathrm{tot}}\left(t\right)=\;\sum_{i=1}^{n}\left(\Delta \varepsilon_{D1}^{i}+\Delta \varepsilon_{D2}^{i}\right).$$

In the present work, we used the input data shown in Table 3.

## 3. Results

**Figure 9:**
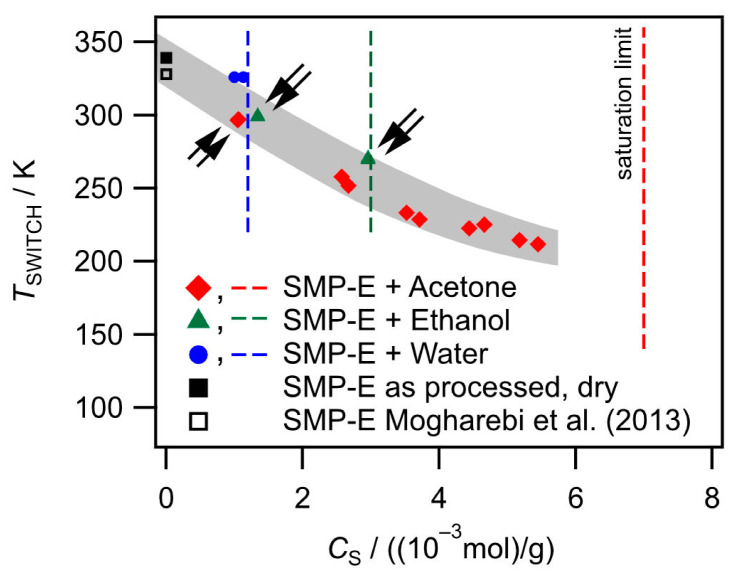
*T*_SWITCH_ from all as-processed and thermally exposed material states studied in the present work.

*Influence of solvent uptake on T_SWITCH_:* We first compile the temperatures *T*_SWITCH_ from all experiments performed in the present work as a function of the solvent concentrations from Table 2. Two data points for as-processed SMP-E are included, one which was reported by Mogharebi et al. [37] and the result presented in Figure 4 for the as-processed material studied in the present work. The data are listed in Table 4 and are presented in Figure 9. 

Figure 9 shows how the concentrations *c*_S_ of acetone, ethanol, and water in SMP-E affect *T*_SWITCH_. The black squares in Figure 9 represent *T*_SWITCH_ of SMP-E prior to chemical exposure (full square: present work; empty square: result from Mogharebi et al. [37]). The red diamonds, green triangles, and blue circles represent data pairs, which were obtained for exposure in acetone, ethanol, and water as indicated. All experiments yielded well reproducible data. The small double arrows indicate that two tests were performed which yielded data points that cannot be distinguished. Vertical dashed lines indicate the saturation levels of SMP-E for acetone (red), ethanol (green), and water (blue). Figure 9 clearly shows that increasing levels of solvent concentrations result in decreasing temperatures *T*_SWITCH_. The lowest value of *T*_SWITCH_ = 210 K (approximately 120 K below the value of the as-processed material) was observed for acetone (lowest data point on the right). A gray scatter band indicates a general tendency; *T*_SWITCH_ decreases with increasing solvent concentration.

**Figure 10:**
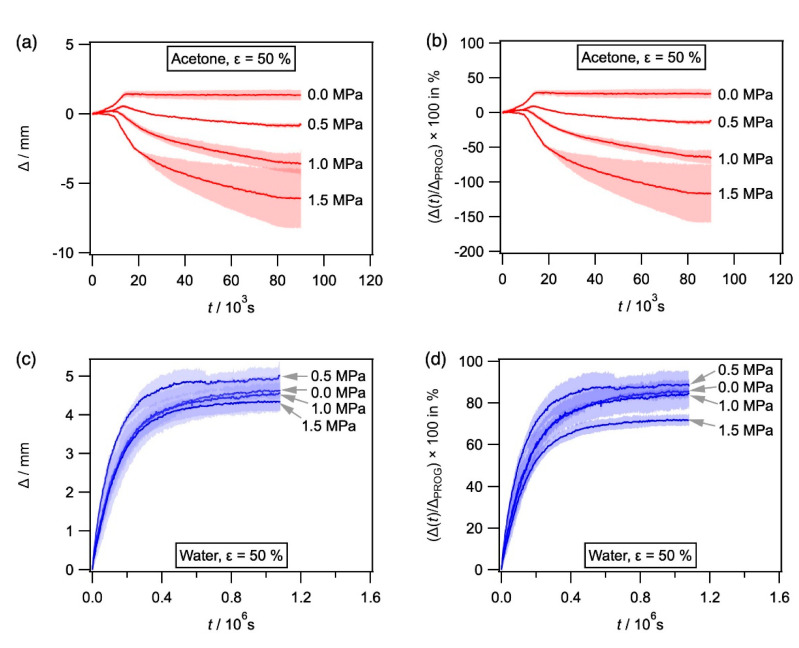
Evolution of Δ with time for SMP-E specimens programmed to 50% and exposed to acetone and water (unconstrained and with varying end loads). (**a**,**b**) Exposure to acetone. (**c**,**d**) Exposure to water. (**a**,**c**) Δ in mm plotted as a function of time in s. (**b**,**d**) (Δ(*t*)/Δ_PROG_) × 100 in % vs. time in s.

*Kinetics of constrained and unconstrained shape recovery:* We now present the results from our measurements of unconstrained and constrained recovery, which were described above. The results for SMP-E which was programmed to 50% programming strain and exposed to ethanol were presented in Figure 7, and the corresponding results obtained for exposure to acetone and water are presented in Figure 10. In Figure 10a (acetone) and Figure 10c (water), displacement Δ(*t*) in mm is plotted as a function of time in s. Figure 10b (acetone) and Figure 10d (water) show the percentage of programming recovered. No shape recovery was observed with acetone as a solvent for an end load corresponding to 1.5 MPa. In the case of water as a solvent, the curves do not exhibit a clear maximum. Instead, the recovery strains increased throughout. Therefore, maximum shape recovery was taken as 99% of the final recovery strain (no arrows shown).

**Figure 11:**
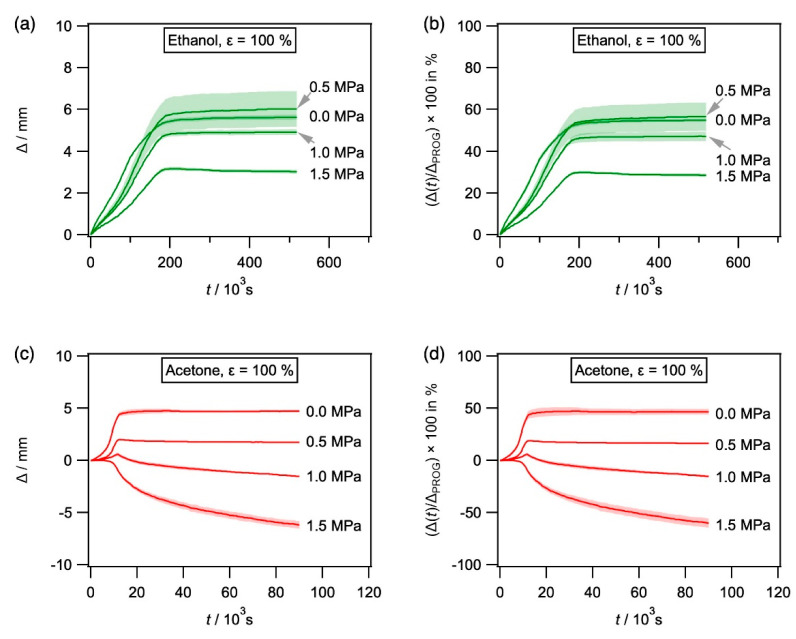

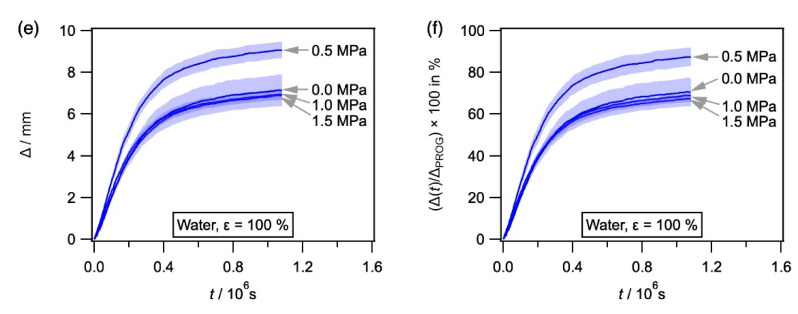
Evolution of Δ with time for SMP-E specimens programmed to 100% and exposed to acetone, ethanol, and water (unconstrained and with varying end loads). (**a**,**b**) Exposure to ethanol. (**c**,**d**) Exposure to acetone. (**e**,**f**) Exposure to water. (**a**,**c**,**e**) Δ in mm plotted as a function of time in s. (**b**,**d**,**f**) (Δ(*t*)/Δ_PROG_) × 100 in % vs. time in s.

Results obtained for SMP-E programmed to a programming strain of 100% during exposure to acetone, ethanol, and water are shown in Figure 11, where the same types of plots were used as in Figure 7 and Figure 10. As can be seen in Figure 11, it is rare that the recovery-time curves show maxima. These are marked by arrows. In all other cases, recovery strains were determined as 99% of the final recovery value. In the case of acetone as a solvent with 1.5 MPa end load, no recovery was observed.

In Figure 13, we show the data which were measured for the three solvents during unconstrained recovery of specimens programmed to 50% and 100% in one plot of (Δ(*t*)/Δ_PROG_) × 100 in % vs. time in s. The data, which were also part of Figure 7, Figure 10 and Figure 11 (with different time scales on the *x*-axis), represent mean values from three experiments (scatter bands presented in Figure 7, Figure 10 and Figure 11 are not shown). Figure 13 allows appreciating that, in the case of unconstrained recovery, all solvents triggered actuation. It can be seen that acetone was fastest but only provided a small effect. The highest effects were obtained for water, albeit taking a long time to become effective. The ethanol data are in between. Figure 13 also shows that while, in case of ethanol and water, the specimens which were programmed to 50% showed the better shape recovery, the 100% programmed specimens in acetone outperformed the 50% specimens in terms of exploitable stroke in mm.

**Figure 12:**
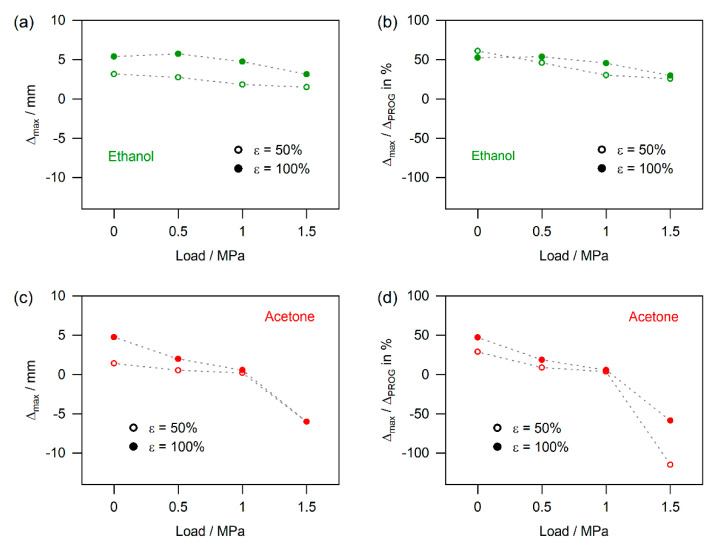

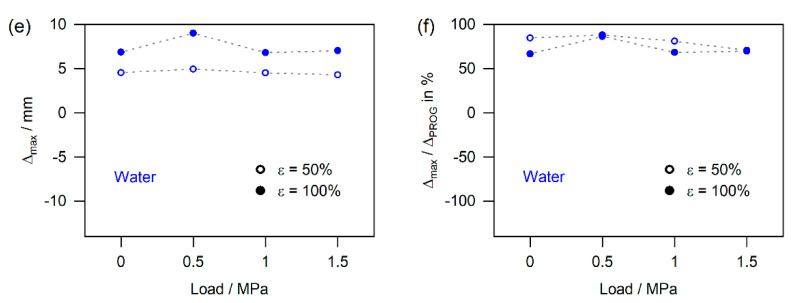
Effect of end loads on actuation. Stresses of 0 (i.e., unconstrained recovery), 0.5, 1.0, and 1.5 MPa are considered. (**a**,**c**,**e**) Stroke Δ_max_ as a function of loading stress. (**b**,**d**,**f**) Percentage of recovered programming strain as a function of loading stress. (**a**,**b**) Ethanol (data points represent the positions which are highlighted by the black arrows in Figure 7). (**c**,**d**) Acetone (where local maxima are observed: coordinates of maximum are presented; in the absence of maxima, 99% of the end value is given). (**e**,**f**) Water (99% of the end value is shown).

Figure 12 shows how end loads expressed as uniaxial average stresses acting in the specimens affect the amount of maximum shape recovery expressed in terms of stroke Δ_max_ and in terms of the part of the programming strain which was recovered in % (Δ_max_/Δ_PROG_). The data presented in Figure 12 were taken from the continuous measurements reported in Figure 7, Figure 10 and Figure 11. In the case of ethanol shown in Figure 12a,b, the data represent the maxima which are marked with arrows in Figure 7. The data for acetone exposure are presented in Figure 12c,d. The same procedure was followed as for ethanol, where maxima could be identified in the continuous curves. In the absence of maxima, 99% of the final value is plotted. For the water results, where continuous contraction was observed throughout, 99% of the end value is plotted (Figure 12e,f).

**Figure 13:**
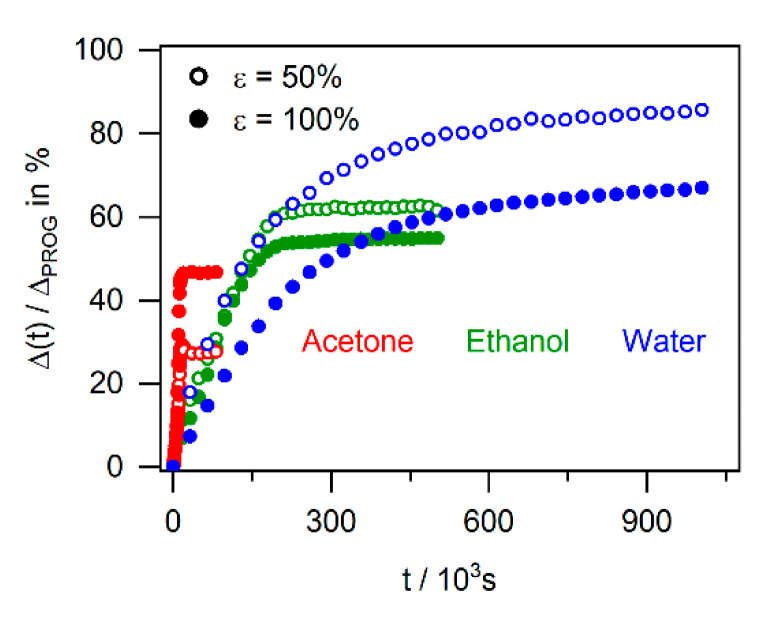
Direct comparison of chemical actuation kinetics of SMP-E. Evolution of (Δ(*t*)/Δ_PROG_) × 100 in % with time in s for unconstrained recovery triggered by acetone, ethanol, and water. For details, see text.

While Figure 12 compiles the achievable effects for the different experimental conditions without considering kinetic aspects, Figure 13 allows appreciating the differences in speed of actuation. It shows how the recovered strain normalized by the programming strain evolves under all unconstrained conditions (*σ* = 0 data from Figure 7, Figure 10 and Figure 11). As was outlined above, each point in Figure 13 represents a mean value of three experiments. It can be clearly seen that unconstrained actuation triggered by acetone (red data points; beginning of plateau: *t* ≈ 20 × 10^3^ s) is 30 times faster than the actuation caused by water exposure (blue data points; better part of recovery reached after *t* ≈ 600 × 10^3^ s). The experiments performed in ethanol (green data points) lie in between. Doubling the programming strain did not affect this finding. Figure 13 also shows that while, in the case of actuation triggered by water and ethanol, a programming strain of 50% was more effectively recovered than a programming strain of 100% (empty symbols above full symbols), the opposite was observed for acetone.

**Figure 14:**
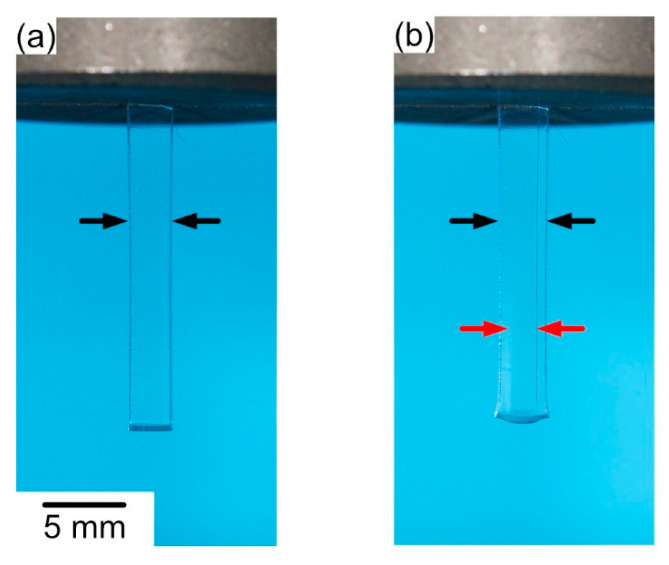
SMP-E (100% programming strain) in acetone. (**a**) Shortly after immersion. (**b**) After 3600 s: sharp diffusion front marked by red arrows. Thickness of diffusion layer: 0.65 mm.

In the case of unconstrained recovery triggered by acetone, a sharp front which separates SMP-E regions with and without solvent moved into the material (Figure 14). Such visible advancing sharp boundaries are commonly observed during swelling of glassy polymers [49]. 

## 4. Discussion

**Figure 15:**
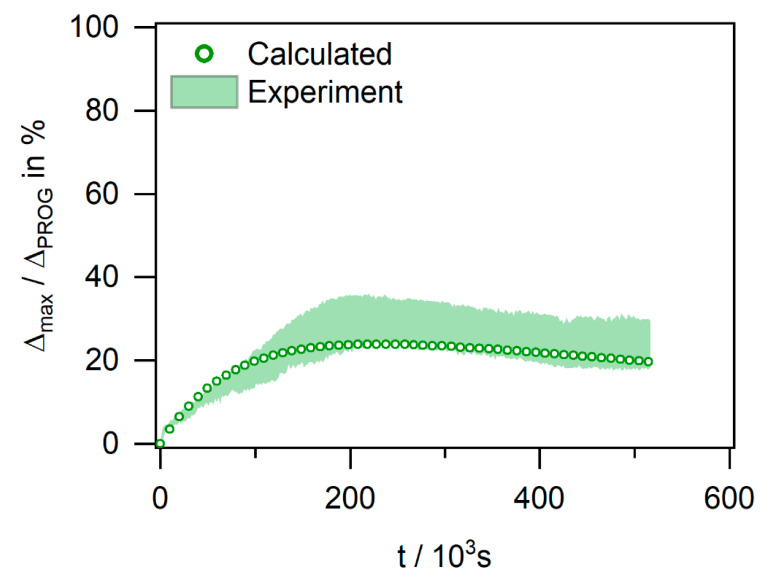
Experimental (green scatter band) and calculated (green circles) evolution of recovery strain with time for SMP-E. Programming strain: 50% ($\Delta_{\mathrm{PROG}}^{*}$ = 30%). Solvent: ethanol. End load: 1.0 MPa. Rheological model: Lethersich [48]. Model: Figure 8a. Input parameters: Table 3.

*Micromechanical interpretation of chemically triggered actuation:*Figure 15 shows the experimental scatter band of data (presented as a green data trend) of the experiment of the SMP-E specimen which was programmed to 50% and exposed to ethanol under an end load of 1.0 MPa (experiment marked with red dashed boxes in Figure 7). Figure 15 also shows the predictions of the micromechanical spring dashpot Lethersich model [48] described above. Figure 15 shows that there is a reasonable agreement between the experimental results and the model predictions. This shows that a Lethersich model captures the essence of the elementary processes which govern the observed shape recovery and creep phenomena. The model can, thus, serve as a basis to model all experiments performed in the present work in a follow-up study.

*Sharp diffusion fronts and molecular mobility:* While the data shown in Figure 15 suggest that one can use the type of model shown in Figure 8a to qualitatively explain our data, it does not account for the complication associated with the result shown in Figure 14b. As small molecules diffuse into SMP-E, surface regions are affected first. Here, the 1WE starts to operate while the inner part of the specimen is not yet chemically affected and counteracts contraction. This requires further micromechanical and physical analysis. A realistic micromechanical model would have to address the composite character (outer layer and inner region) of the system, where stresses are transferred from softer to harder regions, as has been shown for metallic composite systems [50,51]. Further work is required to treat this aspect in detail, but it is beyond the scope of the present work. The observation in Figure 14b can be used, however, to assess the diffusion data which were published in [31]. The thickness of the diffusion layer after 3600 s was 0.65 mm. This can be used to calculate a diffusion coefficient using the following well-known relationship [52,53]:

(7) $$x^{2}=4\;\times \;D\;\times \;t.$$

With the experimentally measured thickness of the diffusion layer of 0.65 mm and the experimental exposure time, we obtain a diffusion coefficient for acetone in SMP-E of $D_{\mathrm{acetone}/\mathrm{SMP}-E}=2.9\;\times 10^{-11}{\;m}^{2}/s$, which is reasonably close to the value of $D_{\mathrm{acetone}/\mathrm{SMP}-E}=1.5\;\times 10^{-11}\;m^{2}/s$ measured using the weight gain method as reported in [31]. It must be kept in mind that, in the present case, we are close to a specimen edge and the small molecules diffuse into a polymer where chains are stretched after programming, while macromolecules were statistically entangled in our previous study [31]. This may explain the small differences in *D* values. The close agreement between the diffusion data obtained by weight gain measurements [31] and derived from microscopic observations (present study) also allows us to conclude that the dark vertical line in Figure 14b represents indeed a diffusion front. Further work is required to explain the nature of this contrast.

**Figure 16:**
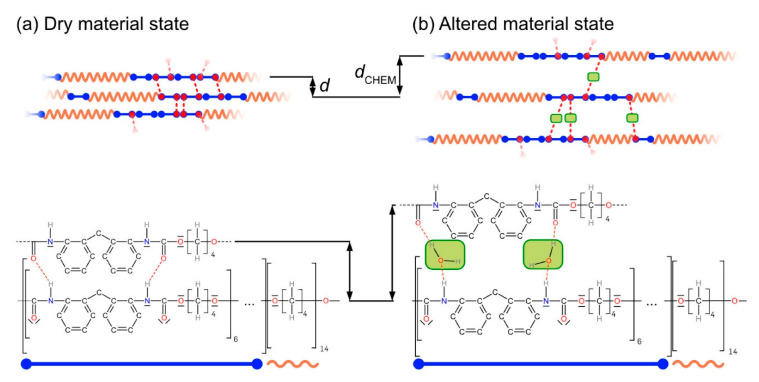
Solvents occupy intermolecular spaces and increase the free volume (schematic illustration). For details, see text.

*Effects of solvents on T_SWITCH_*: From the experimental results presented in Figure 7,Figure 10, Figure 11 and Figure 12, it is clear that not all experiments can be rationalized by the set of data listed in Table 3. Before we discuss this, we must take a closer look at *T*_SWITCH_, which we interpret as the glass temperature of the molecular switch element of SMP-E. Below the glass temperature, polymers can be considered as nonequilibrium glasses because excess free volume is frozen in between kinetically restricted polymer chains. It is well known that, in the glassy state, there is no large-scale macromolecular motion and individual atoms move against constraints imposed by the local atomistic environment, very much like atoms vibrate around equilibrium positions in a crystalline state except that the glassy state has no long-range order (e.g., [47,54]). Above the glass transition temperature, we observe an increase in free volume (e.g., [55,56]). One can now observe liquid-like motion of much longer segments of molecules, characteristic of a highly viscous state. This motion requires more free volume than the short-range excursion of atoms in the glassy state. The results presented in Figure 9 show that the uptake of solvents decreases *T*_SWITCH_ and, thus, the glass temperature of the molecular switch component of SMP-E. In SMP-E, the solvents occupy intra-macromolecular spaces and increase chain-to-chain distances, which in turn causes an increase in free volume. This is illustrated in Figure 16, which uses a part of the structure of the molecular switch as introduced in Figure 2c to rationalize the findings qualitatively. In Figure 16, the intermolecular spacings of the material before and after chemical exposure are referred to as *d* and *d*_CHEM_, respectively. 

From the results presented in Figure 9, one can conclude that, as far as the effect of solvents on *T*_SWITCH_ is concerned, the concentration of the solvent is more important than its chemical nature; after all, all data points fall into a narrow scatter band.

*Chemical actuation of the shape memory effect in SMP-E with different solvents:* However, one needs to consider the chemical nature of solvents and the spatial arrangements of macromolecules to fully account for the chemical actuation phenomena observed in the present study. When discussing the time-dependent plastic deformation of a complex co-polymer such as SMP-E, it is not easy to rationalize plastic deformation on the basis of specific kinds of molecular motion, as Robertson suggested for a planar zigzag chain, where an applied stress can help segments to change from low-energy (*trans*) to higher-energy (*cis*) positions [47,57]. It seems reasonable to assume that the presence of polar groups in SMP-E increases intermolecular bond strength and increases the resistance to plastic flow [58]. In the present study, we have three types of behavior.

First, there are cases where the solvents lower *T*_SWITCH_ and the exposed specimens contract. This is the case when SMP-E is exposed to water (for both programming strains and all end loads considered) (Figure 10c,d and Figure 11e,f). This is also observed for SMP-E exposed to ethanol for end loads below 1.0 MPa for both programming strains (Figure 7a,b and Figure 11a,b). As a striking new result, it is found that, for these two solvents, the recovered strain is higher under a small end load (corresponding to 0.5 MPa) than in the absence of an end load. For water, this is observed for both programming strains (Figure 10c,d and Figure 11e,f). In the case of ethanol, this effect is observed for 100% programming strain (Figure 11a,b).

Second, there are conditions where the specimen first contracts and then, after having reached a maximum contraction, expands. This behavior was reproduced in our simplified rheological model shown in Figure 15. We observe this behavior for ethanol exposure for a 50% programming strain and end loads of 1.0 and 1.5 MPa (Figure 7a,b) and for a programming strain of 100% and an end load of 1.5 MPa. It is also observed for acetone exposure after programming to 50% and an end load of 0.5 MPa (Figure 10a,b) and after programming to 100% and end loads of 0.5 and 1.0 MPa (Figure 11c,d).

Third, we observe cases where the main specimen response is an elongation in the direction of the loading force. This is observed for SMP-E programmed to 50% and 100% exposed to acetone under end loads of 1.5 MPa. Consulting the rheological model from Figure 8a, we can attribute these three behaviors to the viscosities of the two dashpot elements. The first and third cases correspond to situations where $\eta_{D2}\;\gg \;\eta_{D1}$ (only contraction, no elongation) and $\eta_{D2}\;\ll \;\eta_{D1}$ (only expansion, no contraction). It seems reasonable to assume that, in the second case, $\eta_{D1}\leq \;\eta_{D2}$ and the two viscosities have similar orders of magnitude. In the first case, a small end load can promote strain recovery, and further work is required to clarify this intriguing behavior.

*Effect of programming strains and end loads on chemically triggered SMP-E actuation:* The results reported Figure 12 show that, when one focuses on strokes Δ_max_, one can obtain higher displacements for higher programming strains. As can be seen for ethanol exposure in Figure 12a, the strokes obtained for 100% programming strain (full green symbols) are higher throughout than the displacements associated with 50% programming strain (empty green symbols). The differences in stroke are close to a factor of two. However, when considering recovery strains, the two material states do not strongly differ (Figure 12b). Thus, all data points for ethanol exposure (empty green circles: 50% programming strain, full green circles: 100% programming strain) almost fall on one common master curve. This suggests that, for ethanol, we always recover about half of the programming strain. As can be clearly seen in Figure 12c,d, acetone does not qualify for chemical actuation. It can only trigger unconstrained recovery and recovery under small end loads. For end loads above 0.5 MPa, exposure to acetone results in SMP-E creep in the direction of the applied load. This suggests that the softening associated with the uptake of acetone not only allows the molecular switch to free the stretched chains, but reacts with the macromolecules in a way which strongly decreases creep resistance (i.e., strongly decreases the viscosity of the dashpot element D2 in our rheological model). Surprisingly, water exposure yields the largest strokes. As can be seen in Figure 12e,f, it results in the highest strain recoveries and outperforms ethanol. Moreover, within the time scales considered in the present study (for water: up to 12.5 days), the uptake of water does not lead to superimposed viscoplastic deformation of SMP-E. At the same time, actuation triggered by water requires the longest exposure times. These findings are well in line with the diffusion study which preceded the present work, where it was found that *D*_acetone_ > *D*_ethanol_ > *D*_water_ [31]. Our previous study [31] allowed us to set the experimental conditions for the present work. It has been pointed out that, during creep of polymers, one cannot as easily differentiate between an immediate elastic response and a time-dependent creep period [59,60,61,62], as in the case of metals [50,51,63,64]. Moreover, while it is easy to qualitatively understand creep of polymers, it is difficult to identify the rate-controlling process on a molecular level. It is clear, however, that when solvent uptake results in softening, which not only loosens the locking function of the molecular switch but also weakens the resistance to viscoplastic deformation, shape memory polymer actuation is disabled. 

*Chemical actuation of shape memory polymers:* There has been a large body of work on chemical actuation of the one-way effect in macromolecular systems [5,6,8,11,17,18,20,21,22,23,24,65,66,67,68,69,70]. Most of the work published so far has a strong chemical, biochemical, and processing/application-oriented focus. The present work differs from previous studies in that it takes a basic look at chemical/mechanical interactions. On the basis of a previously published solubility/mobility study [31], we compare chemically triggered actuation considering different solvents, different programming strains, and different end loads. The study, which was part of a larger research program on chemical/mechanical interactions [26], represents a first step toward a quantitative micromechanical model of chemically triggered actuation. Our study takes a look at the use of SMP polymers as chemically triggered actuators [32]. It represents a fundamental investigation in the forefield of technical applications. Our results show that it is possible to exploit chemically triggered SMPs for actuation. Chemically triggered SMP actuators should certainly not be thought of as potential replacements for alternative functional materials such as metallic shape memory alloys [71,72]. They can only handle stresses of the order of 1.0 MPa and react slowly. However, they possess the unique feature that they can react to chemical stimuli, which can be exploited in biochemical and chemical engineering applications. Most importantly, they represent a class of materials that allows gaining further insight into how macromolecular morphology and the uptake and diffusion of solids interact with shape memory polymer programming and actuation. Further work is required to identify chemical substances that allow increasing the speed of actuation without causing irreversible creep processes.

## 5. Highlights and Conclusions

In the present work, we performed a parametric study on chemically triggered shape recovery of shape memory polymer actuators made from ESTANE 75DT3 (abbreviated as SMP-E). We exposed SMP-E to chemical environments consisting of acetone, ethanol, and water. The uptake of solvents resulted in a decrease in the glass temperature *T*_SWITCH_ of the molecular switch. The solvents entered SMP-E at different rates (acetone > ethanol > water). In the absence of an end load, all three solvents triggered shape recovery in time scales ranging from a few hours (acetone) to 300 h (water). Water showed the largest recovery strain, whereas acetone showed the lowest recovery strain, and the behavior of ethanol was found to be in between. All three solvents triggered unconstrained shape recovery. They differed strongly in the way they acted when the SMP-E actuator operated against an end load. A simple rheological spring dashpot model of Lethersich type allowed rationalizing strain time reactions, which describe shape memory polymer actuation. Unlike ethanol and water, acetone molecules softened the material too much, such that all mechanical integrity was lost. Larger actuator strokes could be obtained when using larger programming strains. However, when comparing programming strains for 50% and 100%, the normalized recovered strains were similar. Chemically triggered SMP actuators should not be thought of as potential replacements for alternative functional materials such as metallic shape memory alloys. They can only handle stresses of the order of 1.0 MPa and show a slow actuation response. However, they possess the unique feature that they react to chemical stimuli, which can be exploited in biochemical and chemical engineering applications. Moreover, and most importantly, they represent a class of materials that allows gaining further insight into how macromolecular morphology and the physical consequences associated with the uptake of solvents affect shape memory polymer programming and actuation.

## Figures and Tables

**Table 1:** Exposure times *t*_i_ for solvent uptake considered in the present work (in 10^3^ s).

Solvent/Exposure Times *t*_i_	*t*_1_	*t*_2_	*t*_3_	*t*_4_	*t*_5_
Acetone	1.08	7.20	14.40	27.00	90.00
Ethanol	86.40	518.40	-	-	-
Water	1368.00	-	-	-	-

**Table 2:** Molecular weights of solvents, exposure times, results from weight measurements, and resulting concentrations.

**Solvent**	***M_S_* (g/mol)**	***t_i_* (10^3^ s)**	***m*_0_ (g)**	*m*_CH_ (g)	*m*_DMTA_ (g)	*c*_S_ (10^−3^ mol/g)
Acetone	58.08	1.08	0.7683	0.8156	0.8156	1.06
			0.7721	0.8191	0.8190	1.05
		7.20	0.7714	0.8909	0.8865	2.57
			0.7722	0.8919	0.8918	2.67
		14.40	0.7707	0.9392	0.9366	3.71
			0.7692	0.9349	0.9265	3.52
		27.00	0.7683	0.9895	0.9763	4.66
			0.7648	0.9761	0.9620	4.44
		90.00	0.7623	1.0348	1.0034	5.45
			0.7662	1.0214	0.9969	5.18
Ethanol	46.07	86.40	0.7774	0.8255	0.8255	1.34
			0.7703	0.8177	0.8178	1.34
		518.40	0.7582	0.8618	0.8615	2.96
			0.7592	0.8636	0.8628	2.96
Water	18.02	1368.00	0.7687	0.7858	0.7844	1.13
			0.7745	0.7893	0.7884	1.00

**Table 3:** Input data used in the rheological model shown in Figure 8. Calculated evolution of strain: Figure 15.

*E*_S_ (GPa)	*c*_1_ (m^2^/Ns)	*c*_2_ (m^2^/Ns)	Load (MPa)	*ɛ*_S_
6.5	2.0 × 10^−15^	2.0 × 10^−13^	−1.0	0.30

**Table 4:** Compilation of solvent concentrations (for as-processed material: 0) and corresponding temperatures *T*_SWITCH_ of as-processed and chemically exposed SMP-E material states (* in 2013, Mogharebi et al. [37] used the same granulate as we used in the present work, albeit without drying steps; it seems likely that their material took up some humidity).

Solvent	*c*_S_ (10^−3^ mol/g)	*T*_SWITCH_ (K)
SMP-E + acetone	1.06	297.2
	1.05	296.6
	2.57	257.9
	2.67	251.8
	3.71	228.7
	3.52	233.4
	4.66	225.3
	4.44	222.6
	5.45	211.8
	5.18	214.7
SMP-E + ethanol	1.34	299.4
	1.34	299.1
	2.96	270.7
	2.96	269.6
SMP-E+ water	1.13	326.2
	1.00	326.0
SMP-E	as processed	339.1
SMP-E [37]	as processed *	328.0

## Data Availability

The data presented in this study are available on request from the corresponding author.
